# Delivery of Marker‐Free DNA to Plant Genome by the Transgenic Selection‐Associated Fragment Elimination (T‐SAFE) System

**DOI:** 10.1002/pld3.70046

**Published:** 2025-02-05

**Authors:** Yi Yang, Huan Chang, Leiwen Pan, Dongbei Guo, Shun Peng, Ting Mao, Yuehui Zhang, Shui Wang

**Affiliations:** ^1^ Shanghai Collaborative Innovation Center of Plant Germplasm Resources, College of Life Sciences Shanghai Normal University Shanghai China

**Keywords:** CRISPR/Cas9, marker‐free DNA delivery, plant genome, transgenic selection‐associated fragment elimination (T‐SAFE)

## Abstract

The presence of a selection marker in transgenic plants has raised public concerns regarding health safety. We have developed a CRISPR/Cas9‐based DNA delivery system termed transgenic selection‐associated fragment elimination (T‐SAFE). The T‐SAFE system comprises four cassettes: the selection marker, CRISPR/Cas9, spacer‐plus‐protospacer adjacent motif (SP), and the cargo. The first two cassettes, the selection marker and CRISPR/Cas9, are collectively referred to as SCC. The SCC is flanked by two identical SPs derived from the fruit fly *Ebony* gene, which efficiently facilitate the SCC cleavage and subsequently lead to self‐elimination of the selection marker upon integration of exogenous DNA into the plant genome. To inhibit the production of a functional Cas9 protein in bacteria, the IV2 intron of the potato ST‐LS1 gene has been incorporated into the Cas9 gene. Additionally, the Cas9 gene is driven by a reproductive cell‐specific or inducible promoter to avoid SCC cleavage in nonreproductive plant cells. These innovative features allow the T‐SAFE system to achieve an elimination efficiency of the selection marker ranging from 10%–30% in *Arabidopsis* and 5%–8% in rice, with a DNA delivery capacity of approximately 10 kb. This approach offers a safe means for genetically modifying plants.

## 1 Introduction

The advance of modern biotechnology, particularly synthetic biology, has enabled us to genetically modify plants for the desired traits (Chen and Lin 2013). Plant genome could be modified either precisely through homologous recombination and gene editing or randomly integration of the new genes into plant genome (Hanin and Paszkowski 2003; Ramkumar et al. 2020). The frequency of gene targeting (GT) through homologous recombination is often very low in higher plants, which is a barrier for precisely modify plant genome (Lieberman‐Lazarovich and Levy 2011). *Agrobacterium tumefaciens* ‐mediated transfer (T)‐DNA integration into a random position of plant genome is a powerful tool for delivery of a gene into plants (Hwang, Yu, and Lai 2017). However, the selection marker is required for the screening of plant transformants. There is a significant public concern regarding the potential harm to human health associated with selection markers. Over half of the countries in the European Union (EU) have officially implemented bans on the cultivation of genetically modified (GM) crops. The techniques for delivering marker‐free DNA into plant genome have been extensively exploited due to their significant potentials in safe GM applications. Several strategies have been explored, including co‐transformation, transposable element, and site‐specific recombination (Komari et al. 1996; Gleave et al. 1999; Sugita et al. 2000; Cotsaftis et al. 2002; Srivastava and Ow 2004). However, the application of these methods is still tedious and ineffective. A more recent development is the utilization of the CRISPR/Cas9‐based system for elimination of the marker genes (Wang, Wang, and Wang 2019; Tan et al. 2022). In this study, we aim to establish a simplified and efficient CRISPR/Cas9‐based system termed transgenic selection‐associated fragment elimination (T‐SAFE) for delivering marker‐free DNA into plant genome.

## 2 Materials and Methods

### 2.1 Plant Material and Growth Conditions

*Arabidopsis* plants used in this study are of the Columbia (Col‐0) background. The *cpr5‐1* (referred to as *cpr5*) mutant is as described (Peng et al. 2022). Rice plants used in this study are in the background of *Oryza sativa L*. ssp. *Japonica* cv. Zhonghua 11 (ZH11). The growth chamber conditions were set to a temperature of 22°C (for *Arabidopsis*) or 30°C (for rice) and a light intensity of 120 μmol m^−2^ s^−1^ (for *Arabidopsis*) or 200 μmol m^−2^ s^−1^ (for rice), generated by Philips Lifemax Cool White fluorescent bulbs, with a 16‐h light/8‐h dark photoperiod.

### 2.2 Plant Transformation

The *Agrobacterium* strain used for plant transformation was GV3101. The transformation of *Arabidopsis* was performed using the floral dip method as described (Clough and Bent 1998). *Arabidopsis* T0 seeds were sown in soil and, after one week, the herbicide Basta was applied every two days to screen for T1 transformants. The transformation of rice was conducted by Towin Biotechnology (Wuhan, China). For the selection of rice T0 transformants, glufosinate ammonium was used on agar plates. Subsequently, the company planted the T0 transformants and harvested the T1 seeds.

### 2.3 Bacterial Growth Conditions

Bacteria were incubated in 3 mL of LB media at 200 rpm and 37°C for 6 h (for *E. coli* ) or at 200 rpm and 30°C for 12 h (for *A. tumefaciens* ).

### 2.4 Construction

The vector backbone used for all constructs in this study is *pEG100‐SW* (GenBank database under the accession number PP001171), which is derived from the *pEarleyGate 100* (*pEG100*) vector (Table S1) (Earley et al. 2006). The CRISPR‐associated endonuclease Cas9 was from the *Streptococcus pyogenes* , which was driven by *Arabidopsis YAO* (AT4G05410) or *PR1* (AT2G14610) promoter (Lebel et al. 1998; Yan et al. 2015). The single guide RNA (sgRNA) sequence was driven by *Arabidopsis U6–26* (AT3G13855) promoter. The primers used for construction are listed in Table S2.

### 2.5 Immunoblot Analysis

Bacterial pellets or plant ground tissues were boiled in 2x protein loading buffer containing 100 mM Tris‐Cl (pH 6.8), 4% (w/v) SDS (sodium dodecyl sulfate), 0.005% (w/v) bromophenol blue, 10% (v/v) β‐mercaptoethanol and 20% (v/v) glycerol for 10 min. The samples were then separated in an SDS‐PAGE (polyacrylamide gel electrophoresis) gel for immunoblot analysis. The FLAG‐tagged Cas9 proteins were detected using an anti‐FLAG antibody (AE005, ABclonal, Wuhan, China). The antibodies of anti‐α‐tubulin (AF2827, Beyotime, Shanghai, China) and anti‐GAPDH (AF0006, Beyotime) were used as loading controls.

### 2.6 Determination of the SCC Cleavage Efficiency

The SCC cleavage efficiency (%) is determined by calculating the ratio of SCC cleavage events to twice the number of genotyped plants in the T2 generation, and then multiplying by 100. Two pairs of primers, F1 (forward 1)/C‐R (cargo‐reverse) and F2 (forward 2)/C‐R, are utilized for identifying the absence or presence of the SCC.

### 2.7 Thermal Asymmetric Interlaced Polymerase Chain Reaction (TAIL‐PCR)

TAIL‐PCR was conducted as described (Liu et al. 1995). The sequences of gene‐specific primers, including GSP‐R1, GSP‐R2, and GSP‐R3, are listed in Table S2.

### 2.8 Quantitative Reverse‐Transcription Polymerase Chain Reaction (qRT‐PCR)

The qRT‐PCR was performed as described (Peng et al. 2022). Primers used for qRT‐PCR are listed in Table S2.

### 2.9 Statistics

The experiments were conducted in triplicate. The error bar in the graph represents the standard error of the mean (SEM). Data were analyzed by one‐way analysis of variance (ANOVA) with Bonferroni post hoc test or a two‐tailed Student's t‐test. For the ANOVA results, the letter above the bar indicates a statistically significant difference between groups at *p* < 0.05. For the Student's t‐test results, NS, not significant; *p < 0.05; **p < 0.01; ***p < 0.001; ****p < 0.0001.

## 3 Results

### 3.1 The Transgenic Selection‐Associated Fragment Elimination (T‐SAFE) System

**FIGURE 1:**
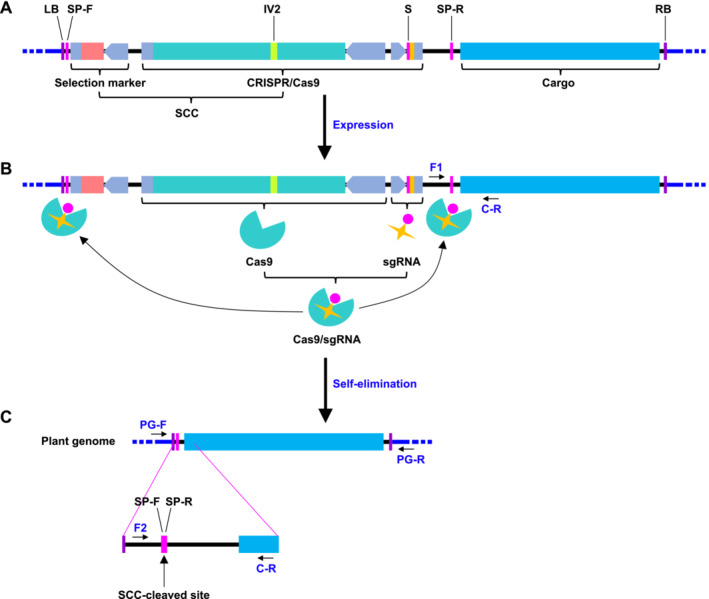
The transgenic selection‐associated fragment elimination (T‐SAFE) system. (A) The T‐SAFE system is composed of four cassettes:the selection marker, CRISPR/Cas9, spacer‐plus‐protospacer adjacent motif (referred to as SP), and the cargo. The selection marker and CRISPR/Cas9 cassettes are collectively referred to as SCC. The CRISPR/Cas9 cassette consists of the Cas9 module and the sgRNA module. The SCC is flanked by two SPs, SP‐F (SP‐Forward) and SP‐R (SP‐Reverse, the reverse sequence of SP‐F), positioned inversely for self‐elimination. Cas9, CRISPR‐associated protein 9; CRISPR, clustered regularly interspaced short palindromic repeats; *IV2*, the second intron of the potato *ST‐LS1* gene; LB, the left border sequence of T‐DNA; RB, the right border sequence of T‐DNA; S, spacer, from the gene of *Drosophila melanogaster* nonribosomal peptide synthetase Ebony; sgRNA, single guide RNA. Detailed information on each component of the T‐SAFE system are described in Figure S1. (B) After the construct is transferred into plant cells and integrated into plant genome, Cas9 and sgRNA are expressed and assembled to form the Cas9/sgRNA complex. The sgRNA then guides the Cas9 nuclease to cleave the target sequences SP‐F and SP‐R. The primer pair, F1 (Forward 1) /C‐R (Cargo‐Reverse), is used to determine the presence of the T‐SAFE construct in plant. (C) If the two CRISPR/Cas9‐targeting sequences SP‐F and SP‐R are cleaved simultaneously, the SCC is eliminated from the genome, while the cargo remains integrated. This process is referred to as transgenic selection‐associated fragment elimination (T‐SAFE). The presence of T‐SAFE in plants is initially identified using PCR and subsequently confirmed through sequencing. The primer pair, F2 (Forward 2)/C‐R, is used to detect the T‐SAFE construct with no SCC. Thermal asymmetric interlaced polymerase chain reaction (TAIL‐PCR) is employed to amplify the flanking plant genome sequence. Based on the TAIL‐PCR results, two primer pairs, PG‐F (Plant Genome‐Forward)/C‐R and PG‐F/PG‐R (Plant Genome‐Reverse), were designed to identify the integration of the genome DNA fragment with the SCC‐cleaved cargo DNA fragment.

The T‐SAFE system comprises four cassettes: the selection marker, CRISPR/Cas9, spacer‐plus‐protospacer adjacent motif (PAM) (referred to as SP), and the cargo (Figure 1A and Figure S1, Table S1). The first two cassettes, the selection marker and CRISPR/Cas9, are collectively referred to as SCC. The Basta resistance gene *BlpR*, encoding phosphinothricin acetyltransferase, is used as the selection marker. The CRISPR/Cas9 cassette consists of the Cas9 module and the sgRNA module. The 20‐base pair spacer (S) sequence (GAACCGGGCAGCCCGCCTCC) of the sgRNA module is derived from the *Drosophila melanogaster* nonribosomal peptide synthetase *Ebony* gene, which was demonstrated to be efficiently cleaved by Cas9 (Ren et al. 2014). The spacer‐plus‐PAM (tgg) sequence is referred to as SP, with SP‐F representing the forward SP sequence (GAACCGGGCAGCCCGCCTCCtgg), and SP‐R representing the reverse SP sequence (ccaGGAGGCGGGCTGCCCGGTTC). Our laboratory's primary research interest is plant immunity. CPR5 is a key negative immune regulator in plant. Therefore, we employed the entire *CPR5* gene as the cargo for the T‐SAFE system, with a size of 5 or 10 kilobases (kb). In addition to heightened immunity, *Arabidopsis cpr5* mutant exhibits pleiotropic morphological phenotypes, such as dwarfism and early senescence (Peng et al. 2022).

Once the T‐SAFE construct is introduced into a plant cell and the T‐DNA is integrated into the plant genome, Cas9 and sgRNA are expressed and form the Cas9/sgRNA complex. The sgRNA guides the Cas9 nuclease to cleave the two targeting sequences, SP‐F and SP‐R. If they are simultaneously cleaved, the SCC cassette will be eliminated, while the cargo is incorporated into the plant genome (Figure 1B). Polymerase chain reaction (PCR) is performed to detect the SCC cleavage. If two copies of the SP sequences flanking the SCC are oriented in the same direction, the DNA polymerase can readily transition from one SP to the other, resulting in the amplification of a fragment of similar size regardless of whether the SCC has been cleaved or not. This phenomenon is referred to as jumping PCR (Paabo, Higuchi, and Wilson 1989). The issue of jumping PCR can be prevented if these two SP sequences are positioned in an inverse manner. To determine if the SCC cassette has been cleaved from its integrated sites, three primers are used: Forward 1 (F1), Forward 2 (F2), and C‐R (Cargo‐Reverse), which are positioned upstream of SP‐R, upstream of SP‐F and downstream of SP‐R, respectively. The primer pair F1/C‐R is used to confirm that the SCC remains intact, while the primer pair F2/C‐R is employed to indicate the SCC cleavage. The PCR products amplified using primer pair F2/C‐R are subsequently sequenced to precisely determine the SCC cleavage. Thermal asymmetric interlaced polymerase chain reaction (TAIL‐PCR) is employed to amplify the flanking plant genome sequence. Based on the TAIL‐PCR results, two primer pairs, PG‐F (Plant Genome‐Forward)/C‐R and PG‐F/PG‐R, were designed to verify the integration of plant genome with the SCC‐cleaved cargo DNA fragment (Figure 1C).

### 3.2 Optimization of the T‐SAFE System

**FIGURE 2:**
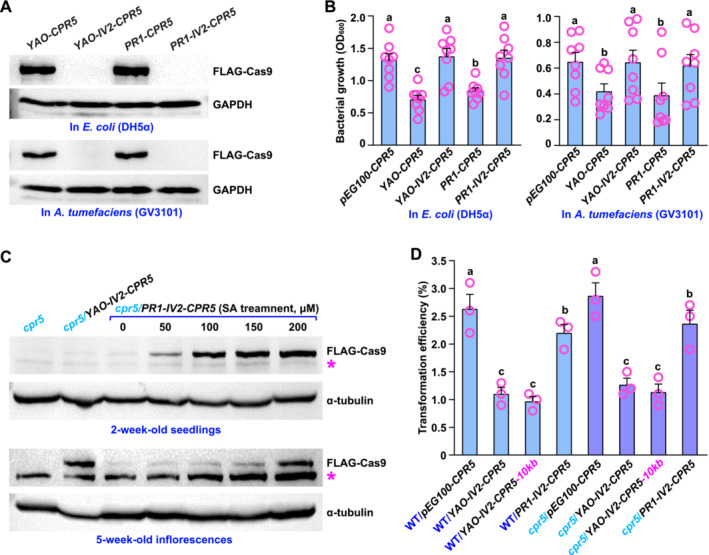
Influence of Cas9 expression on bacterial growth and plant transformation. (A) The total protein extracts from *E. coli* (DH5α) and *A. tumefaciens* (GV3101) strains harboring *YAO‐CPR5* (with the *Cas9* gene), *YAO‐IV2‐CPR5* (with the *IV2‐*integrated *Cas9* gene), *PR1‐CPR5* (with the *Cas9* gene) and *PR1‐IV2‐CPR5* (with the *IV2‐*integrated *Cas9* gene) were analyzed by immunoblotting using an anti‐FLAG antibody to detect the presence of FLAG‐tagged Cas9 proteins. The anti‐GAPDH antibody was used as a loading control. (B) Eight colonies from the plate were picked and incubated. The optical density (OD) at a wavelength of 600 nm was measured to assess the bacterial growth of *E. coli* and *A. tumefaciens* strains carrying *pEG100‐CPR5* (without the *Cas9* gene), *YAO‐CPR5*, *YAO‐IV2‐CPR5*, *PR1‐CPR5*, and *PR1‐IV2‐CPR5*. The experiments were conducted in triplicate (*n* = 3). Bars represent means ± SEM. Statistical differences are indicated with letters (*p* < 0.05, one‐way ANOVA with Bonferroni post hoc test). (C) The total protein extracts of seedlings (two‐week‐old) and inflorescences (5‐week‐old) of *cpr5* (the background), *cpr5*/*YAO‐IV2‐CPR5* and *cpr5*/*PR1‐IV2‐CPR5* (treated with 0, 50, 100, 150 and 200 μM of salicylic acid (SA)) were subjected to immunoblotting using an anti‐FLAG antibody to detect the presence of FLAG‐tagged Cas9 proteins. The anti‐α‐tubulin antibody was used as a loading control. Asterisk (*) indicates a nonspecific band. (D) The transformation efficiency was determined by calculating the percentage of T1 transgenic plants among ~8000 T0 seeds from ~30 T0 plants for constructs of *pEG100‐CPR5*, *YAO‐IV2‐CPR5*, *YAO‐IV2‐CPR5‐10kb* (with a 10‐kb DNA cargo), and *PR1‐IV2‐CPR5* in both wild‐type (WT) and *cpr5* backgrounds. The experiments were conducted in triplicate (*n* = 3). Bars represent means ± SEM. Statistical differences are indicated with letters (*p* < 0.05, one‐way ANOVA with Bonferroni post hoc test).

There are two major limiting factors related to the T‐SAFE system. The first limiting factor is the occurrence of SCC cleavage in bacteria. Our data confirmed the presence of Cas9 proteins in *Escherichia coli* and *A. tumefaciens* , leading to a significant reduction in bacterial accumulation compared to those without the *Cas9* gene (Figure 2A,B). To prevent the expression of a functional Cas9 protein in bacteria, we introduced the second intron, *IV2*, of the potato *ST‐LS1* gene into the *Cas9* gene (Figure 1 and Figure S1 and S2) (Li et al. 2013). As a result, full‐length Cas9 proteins were only detected in bacteria with the *Cas9* gene alone, and not in those with the *IV2*‐integrated *Cas9* gene (Figure 2A). The integration of *IV2* into the *Cas9* gene led to the rescue of bacterial accumulation (Figure 2B).

The second limiting factor is the occurrence of SCC cleavage in the non‐reproductive cells, potentially leading to a reduction in transformation efficiency in plant. The *Cas9* gene is driven by the *YAO* promoter (*P‐YAO*) in the *YAO‐IV2‐CPR5* construct and by the *PR1* promoter (*P‐PR1*) in the *PR1‐IV2‐CPR5* construct. Both *P‐YAO* and *P‐PR1* are derived from *Arabidopsis* (Figure S2). *P‐YAO* highly activates gene expression in reproductive cells. The *pYAO:Cas9* cassette has been shown to achieve efficient gene editing in *Arabidopsis* (Yan et al. 2015). *P‐PR1* is highly responsive to the plant stress hormone salicylic acid (SA) (Lebel et al. 1998). The use of an inducible promoter to drive the *Cas9* gene aims to avoid SCC cleavage in the vegetative tissues of T1 transgenic plants. Western blot analysis revealed that the Cas9 expression in the *YAO‐IV2‐CPR5*‐transgenic plants was prominent in inflorescences, but barely detectable in seedlings. As anticipated, the Cas9 expression in the *PR1‐IV2‐CPR5*‐transgenic plants was minimal in the untreated control, but significantly increased upon SA treatment in both inflorescences and seedlings (Figure 2C).

The transformation efficiency was determined by calculating the percentage of T1 transgenic plants among ~8000 T0 seeds from ~30 T0 plants. In the T1 generation, the transformation efficiencies for the constructs of *pEG100‐CPR5* (containing only the cargo), *YAO‐IV2‐CPR5*, and *PR1‐IV2‐CPR5* in wild type (WT) plants were observed to be 2.63%, 1.07%, and 2.19%, respectively. In *cpr5* mutants, the transformation efficiencies for the corresponding constructs were 2.87%, 1.27%, and 2.34% (Figure 2D). These results suggest that the transformation efficiency is not affected by the plant background (WT and *cpr5*), but the choice of promoter (*P‐YAO* and *P‐PR1*) plays a crucial role. Specifically, the construct with *P‐YAO* exhibits a significantly lower transformation efficiency, less than half of that observed in the control construct. On the other hand, the construct with *P‐PR1* shows a similar transformation efficiency to the control construct. To assess the DNA delivery capacity of the T‐SAFE system, we expanded the size of the cargo to 10 kb in the *YAO‐IV2‐CPR5‐10kb* construct, which is sufficient for most genetic modifications. Our data indicates that delivering the 10‐kb cargo using the T‐SAFE construct in both WT and *cpr5* backgrounds did not significantly differ in transformation efficiency compared to the 5‐kb cargo (Figure 2D).

### 3.3 Delivery of the Marker‐Free DNA Into *Arabidopsis* Genome by the T‐SAFE System

**FIGURE 3:**
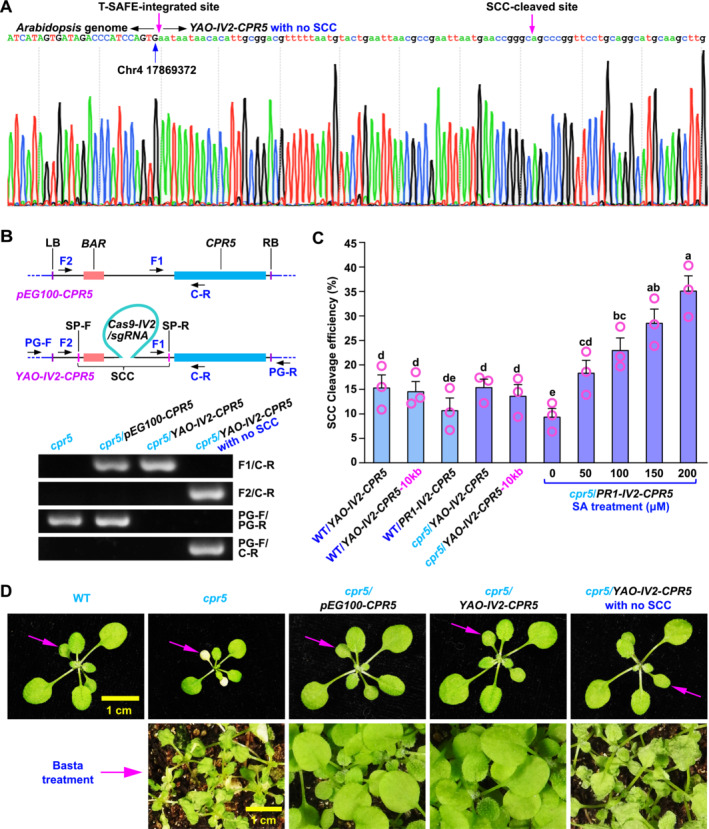
The SCC cleavage efficiency of the T‐SAFE system in *Arabidopsis*. (A) Chromatogram displays the sequencing result of the DNA fragment amplified using PG‐F (Plant Genome‐Forward)/C‐R primers. This fragment encompasses the junction region between *Arabidopsis* genome and the SCC‐cleaved cargo (*YAO‐IV2‐CPR5* with no SCC). (B) The TAIL‐PCR result was validated using standard PCR. Top panel:the schematic diagram illustrating the positions of the primers used. The *YAO‐IV2‐CPR5* construct was derived from the *pEG100‐CPR5* construct and incorporated two additional modules, namely *CRISPR*/*Cas9* and *SP‐F*/*SP‐R*. Two primer pairs, F1/C‐R and F2/C‐R, were used to determine the presence or absence of the SCC, while two primer pairs, PG‐F/C‐R and PG‐F/PG‐R (Plant Genome‐Reverse), were used to identify whether the SCC‐cleaved cargo had integrated into plant genome at the position identified by TAIL‐PCR or not. Bottom panel:the agarose gel image of the PCR result. The *cpr5* mutant served as the plant background. The transgenic plants included *cpr5/pEG100‐CPR5* and two progenies derived from *cpr5/YAO‐IV2‐CPR5*, a plant characterized by TAIL‐PCR, with or without SCC. *BAR*, the Basta resistance gene. (C) The cleavage efficiency was determined by calculating the percentage of T‐SAFE events in ~200 T2 transgenic plants (3 lines per construct) for constructs of *YAO‐IV2‐CPR5*, *YAO‐IV2‐CPR5‐10kb*, and *PR1‐IV2‐CPR5* in both WT and *cpr5* backgrounds. The transgenic plants carrying the *PR1‐IV2‐CPR5* construct in *cpr5* background were treated with 0, 50, 100, 150, and 200 μM of SA. Two primer pairs, F1/C‐R and F2/C‐R, were used to determine the presence or absence of the SCC, respectively. Heterozygous SCC‐cleaved plants are indicated by the amplification of PCR products using both F1/C‐R and F2/C‐R primers. In contrast, a homologous SSC‐cleaved plant is identified when PCR products are detectable only with the F2/C‐R primers. A plant without SSC cleavage is indicated when PCR products are amplified only with the F2/R primers. The experiments were conducted in triplicate (*n* = 3). Bars represent means ± SEM. Statistical differences are indicated with letters (*p* < 0.05, one‐way ANOVA with Bonferroni post hoc test). (D) Top panel:two‐week‐old seedlings of wild type (WT), *cpr5*, *cpr5/pEG100‐CPR5*, *cpr5*/*YAO‐IV2‐CPR5*, and *cpr5*/*YAO‐IV2‐CPR5* with no SCC were photographed for early senescence to assess complementation of the *cpr5* mutant. The arrows indicate cotyledons. Bottom panel:these plants were treated with Basta (glufosinate ammonium, 1:2000, v/v in water) for 2 days and then photographed.

An SCC cleavage refers to the occurrence where the Cas9‐targeting sequences SP‐F and SP‐R are simultaneously cut by Cas9, resulting in the excision of the SCC. In the following generation, we examined the progeny from 8 heterozygous SCC‐cleaved plants identified through PCR and found that all SCC cleavages occurred exclusively in germline cells rather than somatic cells, thereby validating the reliability of this detection method for SCC cleavage (Figure S3A, Table S3). In order to confirm the integration of the SCC‐cleaved cargo into the plant genome, we selected a homologous SCC‐cleaved line and performed TAIL‐PCR to identify the flanking plant genome sequence (Figure S3B and S3C) (Liu et al. 1995). The TAIL‐PCR results demonstrated that the cargo was integrated into the *Arabidopsis* genome between positions 17,869,372 and 17,869,373 on chromosome 4 (Figure 3A). This finding was further validated through standard PCR (Figure 3B).

In the WT background, the SCC cleavage efficiencies of *YAO‐IV2‐CPR5*, *YAO‐IV2‐CPR5‐10kb*, and *PR1‐IV2‐CPR5* were found to be 15.40%, 14.6%, and 10.77%, respectively. In the *cpr5* background, the SCC cleavage efficiencies of *YAO‐IV2‐CPR5* and *YAO‐IV2‐CPR5‐10kb* were determined to be 15.47% and 13.70%, respectively, in T2 generation. Approximately one‐third of the SCC‐cleaved plants (35.3%, 76 out of 215) were found to be homozygous for the SCC‐cleavage trait in the T2 generation of *YAO‐IV2‐CPR5*‐transgenic plants. Additionally, when the *Cas9* gene was driven by *P‐PR1* and exposed to SA at concentrations of 0, 50, 100, 150, and 200 μM in the *cpr5* background, the resulting SCC cleavage efficiencies were observed to be 9.43%, 18.4%, 23.07%, 28.6%, and 35.17%, respectively, in T2 generation (Figure 3C). These data suggest that the SCC cleavage efficiency is not influenced by the plant background (WT and *cpr5*) or the size of the cargo (5‐kb and 10‐kb), but the choice of promoter (*P‐YAO* and *P‐PR1*) plays a critical role. The *YAO* construct showed a lower transformation efficiency of ~1% in the T1 generation, but a higher SCC cleavage efficiency of ~15% in the T2 generation. In contrast, the *PR1* construct exhibited a higher transformation efficiency of ~2% in the T1 generation, but a lower SCC cleavage efficiency of ~10% in the T2 generation. Interestingly, with the *PR1* construct, we observed a significant increase in SCC cleavage efficiency (from 10% to 35%) with the increase in SA concentration (Figures 2D and 3C). These findings suggest that the efficiency of both transformation and SCC cleavage is influenced by the level of Cas9 proteins.

As shown in Figure 3D, all observed defects in the *cpr5* mutant were fully rescued by the *CPR5* gene delivered using the T‐SAFE system. The vector backbone of the T‐SAFE constructs, *pEG100‐SW*, contain a resistance gene to glufosinate ammonium, which is an herbicide and the active ingredient in Basta. Upon Basta treatment, the *cpr5* mutants, which served as the background, were killed, whereas the transgenic plants containing *pEG100‐CPR5* and *YAO‐IV2‐CPR5* survived. As anticipated, the plants from a homologous SCC‐cleaved *YAO‐IV2‐CPR5*‐transgenic line were susceptible to Basta treatment, despite the complementation of the *cpr5* mutant. These data indicate that the cargo was successfully delivered to the plant genome and the associated selection marker was subsequently eliminated.

### 3.4 Delivery of the Marker‐Free DNA Into Rice Genome by the T‐SAFE System

**FIGURE 4:**
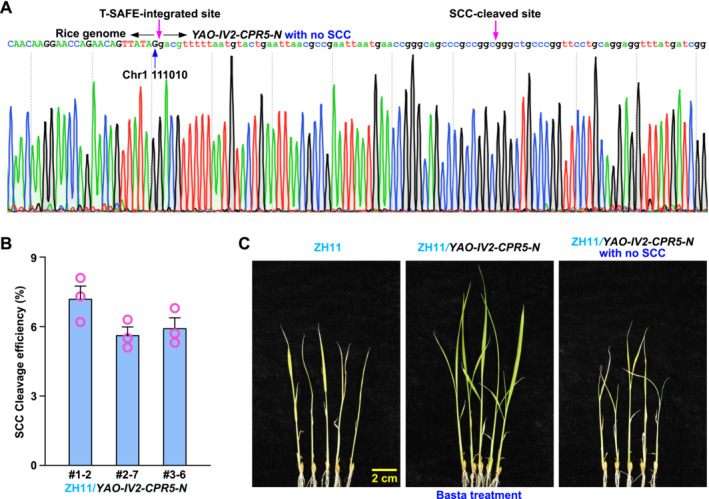
The SCC cleavage efficiency of the T‐SAFE system in rice. (A) Chromatogram displays the sequencing result of the DNA fragment amplified using PG‐F/C‐R primers. This fragment encompasses the junction region between rice genome and the SCC‐cleaved cargo. (B) The cleavage efficiency of the T‐SAFE system in rice was determined by calculating the percentage of T‐SAFE events in ~100 T1 transgenic plants (3 representative lines) of Zhonghua 11 (ZH11)/*YAO‐IV2‐CPR5‐N*. The experiments were conducted in triplicate (*n* = 3). Bars represent means ± SEM. Statistical differences are indicated with letters (*p* < 0.05, one‐way ANOVA with Bonferroni post hoc test). (C) Two‐week‐old seedlings of ZH11 (background) and two‐transgenic plants, ZH11/*YAO‐IV2‐CPR5‐N* with or without SCC, were treated with Basta (1:1000, v/v in water) for 5 days and then photographed.

Our laboratory found that the activity of endogenous CPR5 (564 amino acids) is significantly suppressed by the truncated CPR5‐N, which corresponds to the N‐terminal region of the CPR5 protein (1–340 aa) (Figure S4A,B). CPR5 serves as a key negative regulator of plant immunity (Peng et al. 2022). Therefore, CPR5‐N can be explored to alleviate the suppression of plant immunity. To assess the applicability of the T‐SAFE system in diverse plant species, particularly crops, we introduced the *YAO‐IV2‐CPR5‐N* construct into the rice variety Zhonghua 11 (ZH11). In this construct, *CPR5‐N* is driven by the pathogen‐inducible *P‐PR1* promoter (*PR1:CPR5‐N*) (Figure S4C, Table S1). The TAIL‐PCR results demonstrated that one of the SCC‐cleaved cargos was integrated into the rice genome between positions 111,010 and 111,011 on chromosome 1 (Figure 4A). As shown in Figure 4B, the SCC cleavage efficiency of the T‐SAFE system in rice ranged from 5% to 8% in T1 generation. Approximately one‐third of the SCC‐cleaved plants (30.1%, 34 out of 113) were found to be homozygous for the SCC‐cleavage trait in the T2 generation of *YAO‐IV2‐CPR5‐N*‐transgenic plants. As expected, upon Basta treatment of *YAO‐IV2‐CPR5‐N*‐transgenic plants, those with intact SCC survived, whereas those with cleaved SCC were killed (Figure 4C). The qRT‐PCR analysis confirmed that SA treatment induced the expression of *CPR5‐N* in rice and correspondingly activated the rice defense marker genes, *OsPR1a* and *OsEDS1*, in the *YAO‐IV2‐CPR5‐N*‐transgenic plants to a significantly greater extent than in the ZH11 plants (Figure S4D,E). These findings suggest that the T‐SAFE system could be a universal tool for delivering DNA to the plant genome, while *PR1:CPR5‐N* could be exploited as a general enhancer of plant immunity in response to pathogen infection.

## 4 Discussion

Our findings highlight the significant advantages of the T‐SAFE system as a simplified, high‐capacity, and efficient method for delivering marker‐free DNA to the plant genome. This system achieves high‐capacity through the use of a single sgRNA and two identical SPs, which minimizes the size of the T‐SAFE vector and maximizes the DNA delivery capacity (Figures 1 and S1). In addition, the detection of SCC cleavage is simple and accurate through PCR because the positioning of the two identical SPs in an inverse manner to flank the SCC effectively prevents jumping PCR caused by repeat sequences in the same orientation (Figure 1 and Figure S3A) (Paabo, Higuchi, and Wilson 1989). This system also achieves high efficiency through the utilization of an efficient spacer and an inducible promoter. The Cas9 derived from *Streptococcus pyogenes* has been widely demonstrated as an effective endonuclease, and the spacer, derived from fruit flies, is efficiently cleaved by Cas9 not just in fruit flies but also in *Arabidopsis* and rice (Figures 3 and 4) (Jinek et al. 2014; Ren et al. 2014). The *Cas9* gene expression, driven by the *P‐PR1* promoter, can be extensively and tissue‐specifically induced by SA (Figure 2C). With these innovative features, the SCC cleavage efficiency of the T‐SAFE system ranges from 10% to 30% in *Arabidopsis* and 5% to 8% in rice, with a DNA delivery capacity of ~10 kb, which is sufficient for most genetic modifications (Figures 3C and 4B). While the current elimination efficiency in rice is adequate for implementing T‐SAFE in crops, there is still significant potential for improvement. This can be accomplished by exploring a reproductive cell‐specific promoter that is compatible with different species and can effectively drive the *Cas9* expression.

## Author Contributions

**Shui Wang and Yi Yang:** designed the research, performed the experiments, analyzed the results, wrote the paper. **Huan Chang, Leiwen Pan, Dongbei Guo, Shun Peng, Ting Mao, Yuehui Zhang:** performed the experiments, analyzed the results.

## Conflicts of Interest

The authors declare no conflicts of interest.

## Peer Review

The peer review history for this article is available in the Supporting Information for this article.

## Supporting information


**Figure S1.** Details of the T‐SAFE constructs.
**Figure S2.** The structure of T‐SAFE constructs.
**Figure S3.** The TAIL‐PCR analysis in *Arabidopsis*.
**Figure S4.** The activation of immunity by CPR5‐N and its application in rice using the T‐SAFE system.

**Table S1.** The construction of plasmids used for this study.
**Table S2.** Primers used in this study.
**Table S3.** The inheritance of the SCC cleavage from the T2 generation (with heterozygous SCC cleavage) to T3 the generation.

## Data Availability

The data that support the findings of this study are available from the corresponding author upon reasonable request.
