# Supplementary material for: Delivery of Marker‐Free DNA to Plant Genome by the Transgenic Selection‐Associated Fragment Elimination (T‐SAFE) System
Source: Plant Direct. 2025 Feb 5;9(2):e70046. doi: 10.1002/pld3.70046 (PMC11799591; doi:10.1002/pld3.70046)
Supplement: Supplementary file 1 — Figure S1. Details of the T‐SAFE constructs. Figure S2. The structure of T‐SAFE constructs. Figure S3. The TAIL‐PCR analysis in Arabidopsis. Figure S4. The activation of immunity by CPR5‐N and its application in rice using the T‐SAFE system. [file PLD3-9-e70046-s002.pdf]

Delivery of marker-free DNA to plant genome by the transgenic selection-associated fragment elimination (T-SAFE) system

Yi Yang, Huan Chang, Leiwen Pan, Dongbei Guo, Shun Peng, Ting Mao, Yuehui Zhang, Shui Wang

Shanghai Collaborative Innovation Center of Plant Germplasm Resources, College of Life Sciences, Shanghai Normal University, Shanghai 200234, China

Correspondence should be addressed to S.W. (shuiwang@shnu.edu.cn)

SUPPORTING INFORMATION

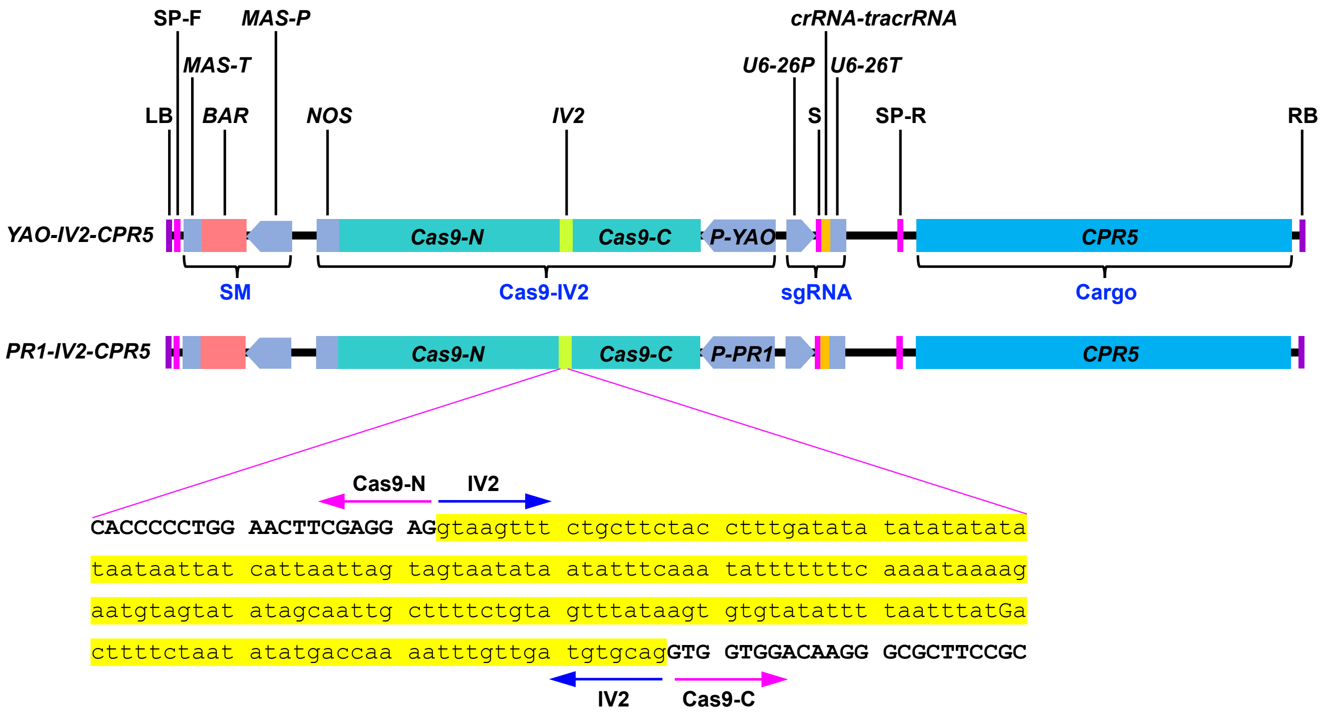
SP-F
MAS-P
crRNA-tracrRNA
MAS-T
U6-26P
U6-26T
LB
BAR
NOS
IV2
S
SP-R
RB
YAO-IV2-CPR5
Cas9-N
Cas9-C
P-YAO
CPR5
SM
Cas9-IV2
sgRNA
Cargo
PR1-IV2-CPR5
Cas9-N
Cas9-C
P-PR1
CPR5
Cas9-N
IV2
CACCCCCTGG AACTTCGAGG AGgtaagttt ctgcttctac ctttgatata tatatatata
taataattat cattaattag tagtaatata atatttcaaa tattttttc aaaataaaag
aatgtagtat atagcaattg cttttctgta gtttataagt gtgtatattt taatttatGa
cttttctaat atatgaccaa aatttgttga tgtgcagGTG GTGGACAAGG GCGCTTCCGC
IV2
Cas9-C

Figure S1 Details of the T-SAFE constructs.

The T-SAFE system is composed of four cassettes: the selection marker (*MAS-P:BAR:MAS-T*), CRISPR/Cas9 (the Cas9 module, *P-YAO* or *P-PR1:Cas9-IV2:NOS-T*; the sgRNA module, *U6-26P:sgRNA:U6-26T*), spacer-plus-protospacer adjacent motif (referred to as SP), and the cargo (the *CPR5* gene). The Basta resistance gene *BIALAPHOS RESISTANCE* (*BAR*), encoding phosphinothricin acetyl-transferase, is used as the selection marker. The spacer (S) sequence of the sgRNA module, GAACCGGGCAGCCCGCCTCC, is derived from the *Drosophila melanogaster* nonribosomal peptide synthetase Ebony gene, which has previously been demonstrated to be high efficiently cleaved by Cas9. This spacer and PAM (tgg) together make up the SP, which serves as the target for CRISPR/Cas9. The SCC is flanked by two SPs, SP-F (SP-Forward, GAACCGGGCAGCCCGCCTCCtgg) and SP-R (SP-Reverse, the reverse sequence of SP-F, ccaGGAGGCGGGCTGCCCGGTTC), positioned inversely for self-elimination. The *Cas9* gene is split by *IV2*, the second intron of the potato *ST-LS1* gene, into two parts (*Cas9-N* and *Cas9-C*, encoding nitrogen- and carbon-terminus the Cas9 protein, respectively). The bases of the *Cas9* gene are denoted in capital letters, while the bases of the intron *IV2* (195 bp) are indicated in lowercase letters (highlighted in yellow). *CPR5*, *CONSTITUTIVE EXPRESSION OF PR GENES 5* (AT5G64930, 4597 bp, 25945277~25949873 on chromosome 5), which is used as a cargo of the T-SAFE system; CRISPR, clustered regularly interspaced short palindromic repeats; Cas9, CRISPR-associated protein 9, which is fused with a FLAG (DYKDDDDK) tag; *Cas9-IV2*, the *IV2*-integrated *Cas9* gene; LB, the left border sequence of transfer (T)-DNA; *MAS-P*, the *MANNOPINE SYNTHASE* (*MAS*) promoter; *MAS-T*, the *MAS* terminator; *NOS*, the *NOPALINE SYNTHASE* (*NOS*) terminator; *P-PR1*, *Arabidopsis PATHOGENESIS-RELATED PROTEIN 1* (*PR1*, AT2G14610) promoter; *P-YAO*, *Arabidopsis YAOZHE* (*YAO*, AT4G05410) promoter; RB, the right border sequence of T-DNA; sgRNA, single guide RNA, including CRISPR RNA (crRNA) and trans-activating crRNA (tracrRNA);

SM, selection marker; *U6-26P*, the *U6-26* (AT3G13855) promoter; *U6-26T*, the *U6-26* terminator.

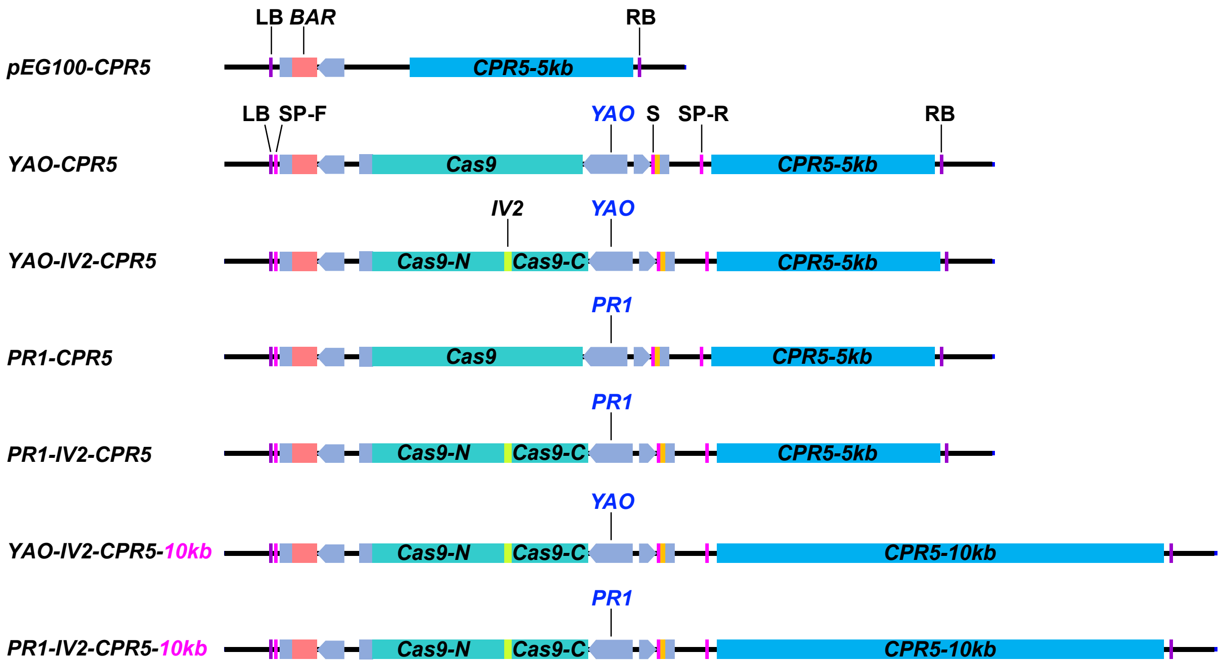

pEG100-CPR5
LB
BAR
CPR5-5kb
RB
YAO-CPR5
LB
SP-F
Cas9
YAO
S
SP-R
CPR5-5kb
RB
YAO-IV2-CPR5
IV2
YAO
Cas9-N
Cas9-C
CPR5-5kb
PR1-CPR5
PR1
Cas9
CPR5-5kb
PR1-IV2-CPR5
PR1
Cas9-N
Cas9-C
CPR5-5kb
YAO-IV2-CPR5-10kb
YAO
Cas9-N
Cas9-C
CPR5-10kb
PR1-IV2-CPR5-10kb
PR1
Cas9-N
Cas9-C
CPR5-10kb

Figure S2 The structure of T-SAFE constructs.

The T-SAFE constructs used in Figure 2 include *pEG100-CPR5*, *YAO-CPR5*, *YAO-IV2-CPR5*, *PR1-CPR5*, *PR1-IV2-CPR5*, *PR1-IV2-CPR5-10kb*, and *YAO-IV2-CPR5-10kb*. Detailed information on each component of the T-SAFE system are described in Figure S1.

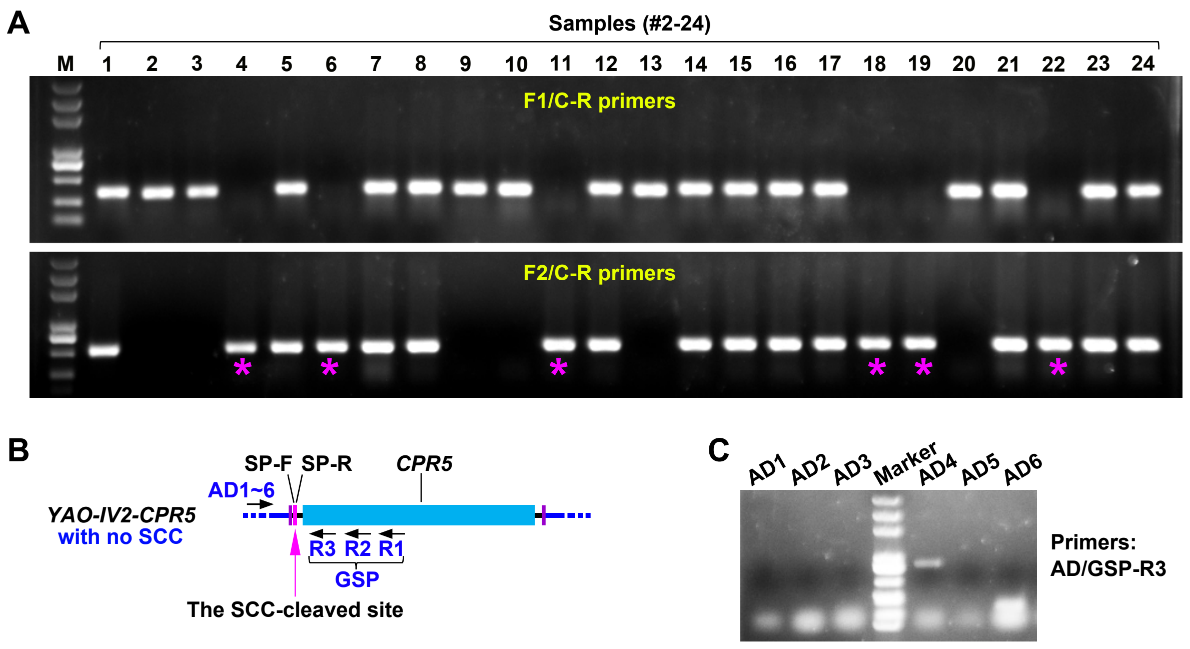
A
Samples (#2-24)
M 1 2 3 4 5 6 7 8 9 10 11 12 13 14 15 16 17 18 19 20 21 22 23 24
F1/C-R primers
F2/C-R primers
B
SP-F SP-R
CPR5
AD1~6
YAO-IV2-CPR5
with no SCC
R3 R2 R1
GSP
The SCC-cleaved site
C
AD1 AD2 AD3 Marker AD4 AD5 AD6
Primers:
AD/GSP-R3

Figure S3 The TAIL-PCR analysis in *Arabidopsis*.

(A) The agarose gel image shows the PCR products of twenty-four T3 progenies derived from a representative T2 *YAO-IV2-CPR5*-transgenic plant (#2-24), which was identified as a heterozygous SCC-cleaved plant. Two pairs of primers, F2/C-R and F1/C-R, were used to determine whether the SCC was cleaved out or not. * indicates the homozygous SCC-cleaved line.

(B) Schematic diagram illustrates the TAIL-PCR method, which involves the use of Arbitrary Degenerate primers 1~6 (AD1~6) and Gene Specific Primer-Reverse 1~3 (GSP-R1~3). PCR was successively performed using three primer pairs: AD/GSP-R1, AD/GSP-R2, and AD/GSP-R3.

(C) The agarose gel image displays the result of TAIL-PCR using the third primer pair AD/GSP-R3.

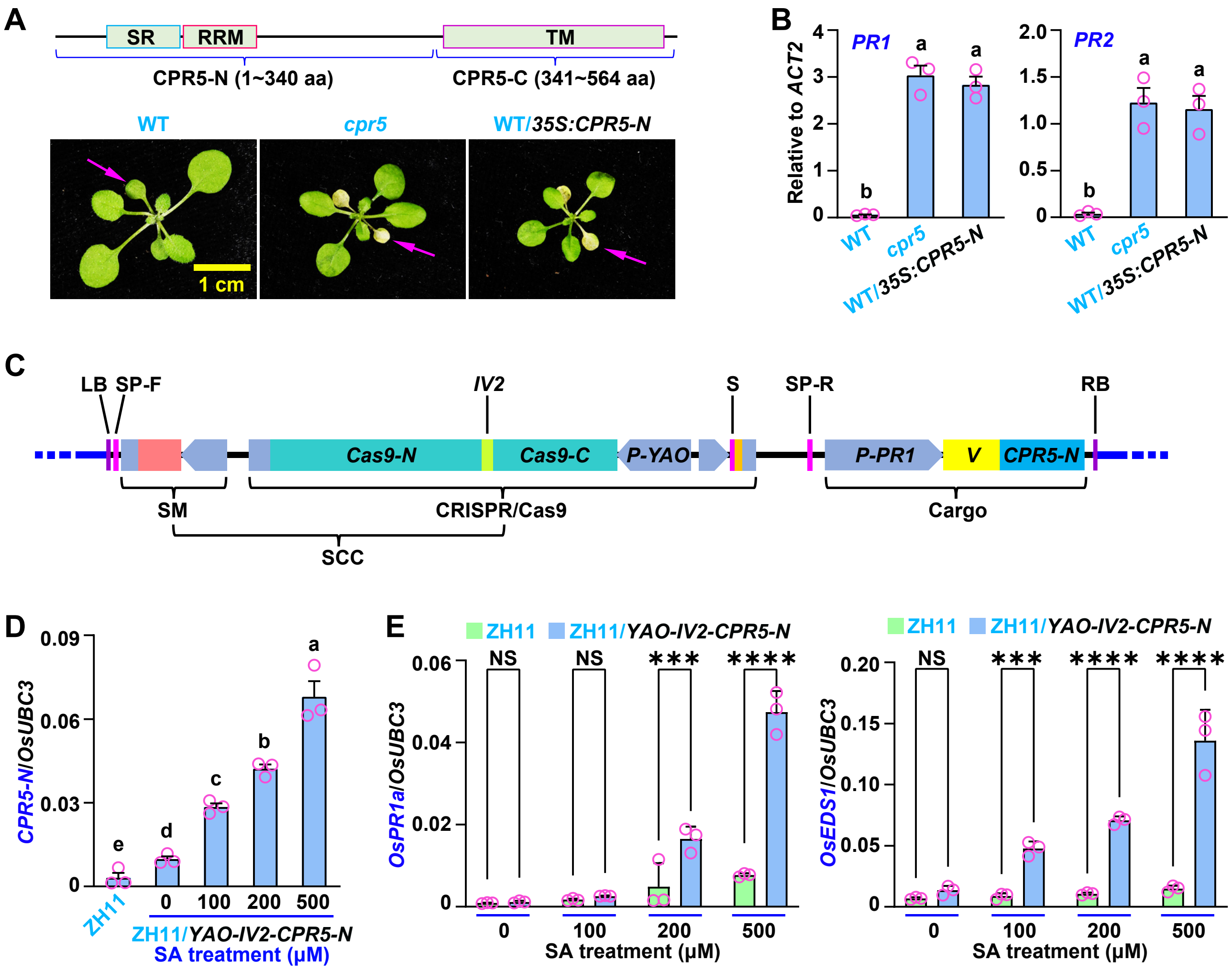
A
SR
RRM
TM
CPR5-N (1~340 aa)
CPR5-C (341~564 aa)
WT
cpr5
WT/35S:CPR5-N
1 cm
B
PR1
PR2
Relative to ACT2
WT
cpr5
WT/35S:CPR5-N
C
LB
SP-F
IV2
S
SP-R
RB
Cas9-N
Cas9-C
P-YAO
P-PR1
V
CPR5-N
SM
CRISPR/Cas9
Cargo
SCC
D
CPR5-N/OsUBC3
ZH11
ZH11/YAO-IV2-CPR5-N
SA treatment (μM)
E
ZH11
ZH11/YAO-IV2-CPR5-N
OsPR1a/OsUBC3
OsEDS1/OsUBC3
NS
***
****
SA treatment (μM)

Figure S4 The activation of immunity by CPR5-N and its application in rice using the T-SAFE system.

(A) Top panel: the structure of CPR5 protein. SR, Serine (S)/arginine (R)-rich domain; RRM, RNA-recognition motif; TM, Transmemebrane domain; CPR5-N, the nitrogen (N)-terminus of CPR5 protein (1~340 amino acid residues); CPR5-C, the carbon (C)-terminus of CPR5 protein (341~564 amino acid residues). Bottom panel: two-week-old wild type (WT), *cpr5* and WT/*35S:CPR5-N* (the *35S* promoter driven *VENUS-CPR5-N* fusion gene, in WT background)-transgenic plants were photographed for early senescence. Cotyledons are indicated (arrows).

(B) The qRT-PCR was carried out on the *Arabidopsis* defense marker genes *PR1* (left) and *PR2* (right) in 12-day-old seedlings of WT, *cpr5*, and WT/*35S:CPR5-N* plants. *ACT2* was used as an internal control. The experiments were conducted in triplicate (n = 3). Bars represent means ± SEM. Statistical differences are indicated with letters (P < 0.05, one-way ANOVA with Bonferroni post hoc test).

(C) Schematic diagram of the *YAO-IV2-CPR5-N* construct. The fluorescent protein VENUS (V) is fused to the N-terminus of CPR5-N. This fusion gene is driven by *P-PR1* (*PR1:CPR5-N*), which has a total length of 3310 bp and serves as the cargo.

(D) The qRT-PCR was carried out on *CPR5-N*, which encodes the N-terminus of *Arabidopsis* CPR5 protein (1~340 aa), in two-week-old ZH11 and ZH11/*YAO-IV2-CPR5-N* plants. The transgenic plants were treated with 0, 100, 200, and 500 µM of SA. *OsUBC3* (Os02g0634800) was used as an internal control. The experiments were conducted in triplicate (n = 3). Bars represent means ± SEM. Statistical differences are indicated with letters (P < 0.05, one-way ANOVA with Bonferroni post hoc test).

(E) The qRT-PCR was carried out on the rice defense marker genes *OsPR1a* (Os07g0129200, left) and *OsEDS1* (Os09g0392100, right) in two-week-old Zhonghua 11 (ZH11) and ZH11/*YAO-IV2-CPR5-N* plants. The plants were treated with 0, 100, 200, and 500 µM of SA. *OsUBC3* (Os02g0634800) was used as an internal control. The

experiments were conducted in triplicate ($n = 3$). Bars are represented as mean ± SEM. Data were analyzed by the two-tailed Student's t-test. NS, not significant; ***, $P < 0.001$; ****, $P < 0.0001$.
