# Supplementary material for: Delivery of Marker‐Free DNA to Plant Genome by the Transgenic Selection‐Associated Fragment Elimination (T‐SAFE) System
Source: Plant Direct. 2025 Feb 5;9(2):e70046. doi: 10.1002/pld3.70046 (PMC11799591; doi:10.1002/pld3.70046)
Supplement: Supplementary file 2 — Table S1. The construction of plasmids used for this study. Table S2. Primers used in this study. Table S3. The inheritance of the SCC cleavage from the T2 generation (with heterozygous SCC cleavage) to T3 the generation. [file PLD3-9-e70046-s001.docx]

**Table S1** **The sequences of plasmids constructed for this study.**

Included constructs are: *pEG100-CPR5*, *PR1-CPR5*, *PR1-IV2-CPR5*, *YAO-CPR5*, *YAO-IV2-CPR5*, *YAO-IV2-CPR5-10k* and *YAO-IV2-CPR5-N*.

| **Plasmid^1^** | **Backbone vector^2^** | **Construction^3^** |
| --- | --- | --- |
| *pEG100-CPR5* | *pEG100-SW* | CPR5 at PmeI |
| *PR1-CPR5* | *pEG100-SW* | *P-PR1:Cas9:NOS-T* in StuI and PacI;  *U6-26P:sgRNA:U6-26T* in PacI and SmaI; SP-F at SacI; SP-R at SbfI; CPR5 at PmeI |
| *PR1-IV2-CPR5* | *pEG100-SW* | *P-PR1:Cas9-IV2:NOS-T* in StuI and PacI; *U6-26P:sgRNA:U6-26T* in PacI and SmaI; SP-F at SacI; SP-R at SbfI; CPR5 at PmeI |
| *YAO-CPR5* | *pEG100-SW* | *P-YAO:Cas9:NOS-T* in StuI and PacI;  *U6-26P:sgRNA:U6-26T* in PacI and SmaI; SP-F at SacI; SP-R at SbfI; CPR5 at PmeI |
| *YAO-IV2-CPR5-10kb or*  *YAO-IV2-CPR5-10kb* | *pEG100-SW* | *P-YAO:Cas9-IV2:NOS-T* in StuI and PacI; *U6-26P:sgRNA:U6-26T* in PacI and SmaI; SP-F at SacI; SP-R at SbfI; CPR5 at PmeI |
| *YAO-IV2-CPR5-N* | *pEG100-SW* | *P-YAO:Cas9-IV2:NOS-T* in StuI and PacI; *U6-26P:sgRNA:U6-26T* in PacI and SmaI; SP-F at SacI; SP-R at SbfI; CPR5-N at PmeI |

^1^The full-length sequences of *pEG100-CPR5*, *PR1-IV2-CPR5*, *YAO-IV2-CPR5* and *YAO-IV2-CPR5-N* are displayed below.

^2^pEG100-SW: GenBank database under the accession number PP001171.

^3^“at”, inserted at a restriction enzyme; “in”, inserted between two restriction enzymes; *Cas9-IV2*, the *IV2-*integrated *Cas9* gene.

**Table S2 Primers used in this study.**

| **Primer name** | **Gene identification** | **Sequence (5’-3’)** |
| --- | --- | --- |
| F1 |  | CTTGAAGTTGAGTATTGGCCGTC |
| F2 |  | GACAACTTAATAACACATTGCGGACGTT |
| C-R |  | GCAATAACATATACTCCCTCCGAATCAG |
| GSP-R1 |  | GTTGGCACTTTGCAGTGAGAGG |
| GSP-R2 |  | GAGCAGCTTGAGCTTGGATCAG |
| GSP-R3 |  | AGCTTGGCACTGGCCGTCGTT |
| ACT2-qPCR-F | AT3G18780 | GGCTCCTCTTAACCCAAAGGC |
| ACT2-qPCR-R |  | CACACCATCACCAGAATCCAG |
| PR1-qPCR-F | AT2G14610 | CTCATACACTCTGGTGGG |
| PR1-qPCR-R |  | TTGGCACATCCGAGTC |
| PR2-qPCR-F | AT3G57260 | CAGATTCCGGTACATCAACG |
| PR2-qPCR-R |  | AGTGGTGGTGTCAGTGGCTA |
| OsJ-UBC3-qPCR-F | Os02g0634800 | CCGTTTGTAGAGCCATAATTGCA |
| OsJ-UBC3-qPCR-R |  | AGGTTGCCTGAGTCACAGTTAAGTG |
| OsJ-PR1a-qPCR-F | Os07g0129200 | GCGAGTTCGTCGAGCAGGTT |
| OsJ-PR1a-qPCR-R |  | GTTGTGCGGGTCCACGAAGT |
| Os-EDS1-qPCR-F | Os09g0392100 | TCAGTTGGATCCCCAGCAA |
| Os-EDS1-qPCR-R |  | TCCCAAGTAATCCACGCAAAC |

**Table S3** **The inheritance of the SCC cleavage from the T2 generation (with heterozygous SCC cleavage) to T3 the generation.**

The SCC cleavage frequency (%) was assessed in the T3 progenies (n = 48) derived from eight heterozygous T2 plants exhibiting the SCC cleavage.

| **Line number of T2 plants** | **T3 progenies with SCC cleavage** | | | | $\boldsymbol{\chi}^{\boldsymbol{2}}$ **test**  **(1:2:1)** |
| --- | --- | --- | --- | --- | --- |
|  | **No cleavage** | **Heterozygous** | **Homozygous** | **Total** |  |
| 1 | 15 | 22 | 11 | 48 | 0.6065 |
| 2 | 14 | 20 | 14 | 48 | 0.5134 |
| 3 | 13 | 21 | 14 | 48 | 0.6731 |
| 4 | 10 | 27 | 11 | 48 | 0.6731 |
| 5 | 15 | 20 | 13 | 48 | 0.4724 |
| 6 | 9 | 26 | 13 | 48 | 0.6065 |
| 7 | 18 | 21 | 9 | 48 | 0.1271 |
| 8 | 13 | 22 | 13 | 48 | 0.8465 |
